# [Retracted] IQGAP1‑siRNA inhibits proliferation and metastasis of U251 and U373 glioma cell lines

**DOI:** 10.3892/mmr.2023.13094

**Published:** 2023-09-18

**Authors:** Bo Diao, Ying Liu, Yi Zhang, Jing Yu, Jun Xie, Guo-Zheng Xu

Mol Med Rep 15: 2074–2082, 2017; DOI: 10.3892/mmr.2017.6257

Following the publication of this paper, it was drawn to the Editor's attention by a concerned reader that the GAPDH control western blotting data shown in Fig. 1C, and other western blotting data included in Figs. 2D and 7C and D, were strikingly similar to data appearing in different form in other articles written by different authors at different research institutes.

Owing to the fact that the contentious data in the above article were already under consideration for publication, or had already been published elsewhere, prior to its submission to *Molecular Medicine Reports*, the Editor has decided that this paper should be retracted from the Journal. The authors were asked for an explanation to account for these concerns, but the Editorial Office did not receive a reply. The Editor apologizes to the readership for any inconvenience caused.

